# Appendix Mucinous Neoplasm

**DOI:** 10.1002/ccr3.72939

**Published:** 2026-06-30

**Authors:** Ping Xiao, Tao Xu, Ming Zhou, ZhenYu Lei

**Affiliations:** ^1^ Department of Surgery Dahua Hospital Xuhui Shanghai China

**Keywords:** diagnosis, laparoscope surgical, low‐grade appendiceal mucinous neoplasms, treatment

## Abstract

A 36‐year‐old female patient accepted the laparoscopic appendectomy; she was diagnosed with low‐grade appendiceal mucinous neoplasm. There are no signs of recurrence after 4 years of follow‐up. We review and discuss the advanced literature on LAMN diagnosis and surgical treatment principles.

## 1 Introduction

Low‐grade appendiceal mucinous neoplasm (LAMN) is an uncommon epithelial tumor of the appendix that grows slowly and produces mucus; the incidence is 0.12 instances per million persons [1]. It can be difficult to diagnose because of the lack of typical symptoms. Acute appendicitis and right lower quadrant pain are typically described as difficult cases, and diagnoses are commonly made intraoperatively and histologically. Acute appendicitis and right lower quadrant pain are typically described as difficult cases, and diagnoses are commonly made intraoperatively and histologically, incorrectly identified as ovarian tumors in female patients when mucinous neoplasm are not recognized and removed before appendiceal rupture, pseudomyxoma peritonei (PMP) may result. There are no relevant guidelines for the diagnosis and treatment of LAMN due to its low incidence rate; most articles are case reports. We reviewed the LAMN treatment principle and further analyzed the advantages of laparoscopic surgery (LS) for patients in this case report. The purpose is to help surgeons understand when we need to perform right hemicolectomy and to resect the neoplasia totally and carefully in the process of practice if we realize that patients may be suffering from PMP at risk because of appendiceal rupture.

## 2 Case History

**FIGURE 1:**
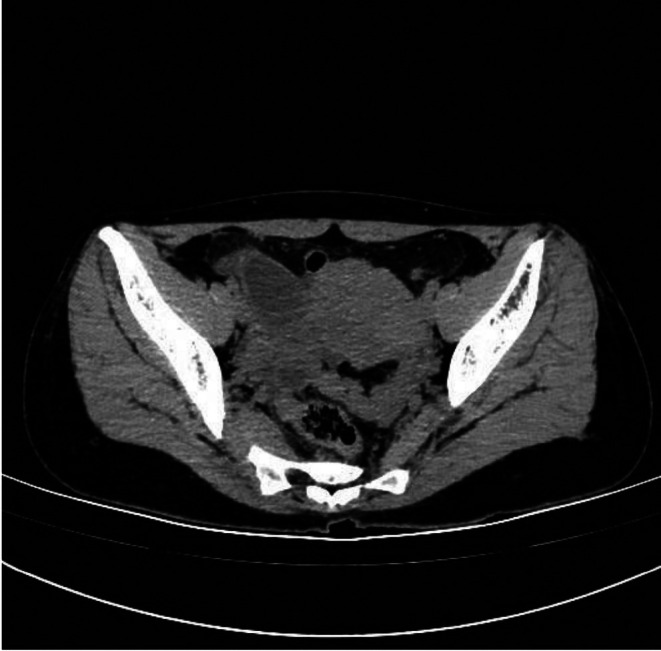
Computed tomography showed cystic low‐density lesions in the area of the right lower abdomen.

A 36‐year‐old young woman presented with a 1‐day history of fixed acute right lower quadrant pain. Acute pain was noted in the right lower abdomen; ultrasonography revealed a large cystic mass measuring 13.0 × 2.9 cm in the right pelvic cavity with a clear distinction from the surrounding tissue. Enhanced computed tomography (CT) scans showed low‐density cystic lesions in the right lower abdomen. The size of the tumor was approximately 13 × 2.9 cm, and the CT value was 10 HU. Mucinous tumors derived from the appendix should be considered first, and tubal abscesses should be excluded (Figure 1). The CT report demonstrated that the margin was blurred, with a suspected connection to the appendix.

## 3 Differential Diagnosis, Investigations and Treatment

**FIGURE 2:**
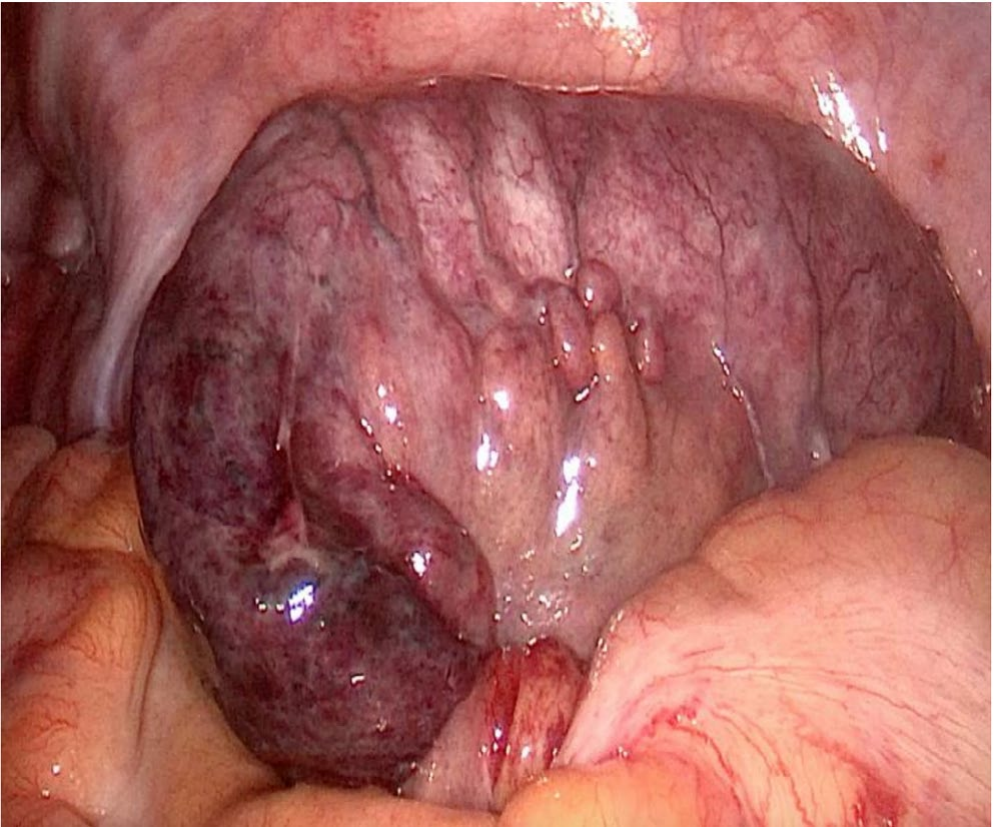
The appendix root torsion was approximately 540°.

It is extremely difficult to distinguish LAMN from a gynecological ovarian cyst; sometimes, they are often misdiagnosed as one another. For our patient, she was misdiagnosed of gynecological ovarian cyst 1 year before she accepted the operation. When the patient accepted the laparoscopic surgical treatment, we invited the experts in gynecology to analyze the patient's detailed information together. We found nearly 100 mL of a pale yellow, transparent liquid not mucus in the pelvis during the operation. The appendix was swollen, and the appendix body showed necrosis and ischemia due to torsion of the root at about 540°, because of the tumor distal end of the appendix is significantly thicker than the normal root, resulting in significant ischemia of the distal end caused by torsion of the root, the capsule was complete, and further exploration of the right ovary showed no signs of distal metastasis or tumor deposits (Figure 2). A laparoscopic inspection performed during the procedure showed no signs of peritoneal pseudomyxoma metastases, and the tumor was entirely excised.

**FIGURE 3:**
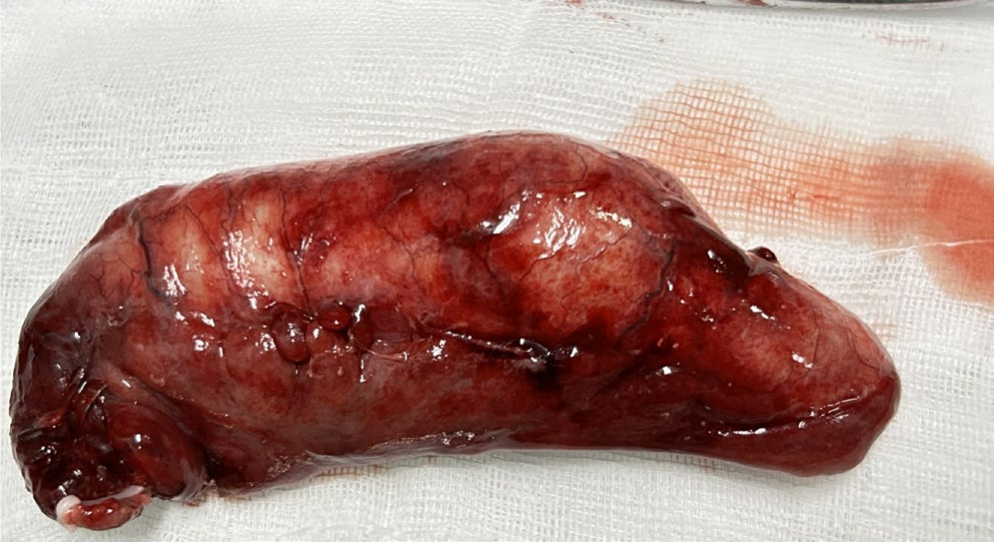
The appendix was resected by laparoscopic surgery.

**FIGURE 4:**
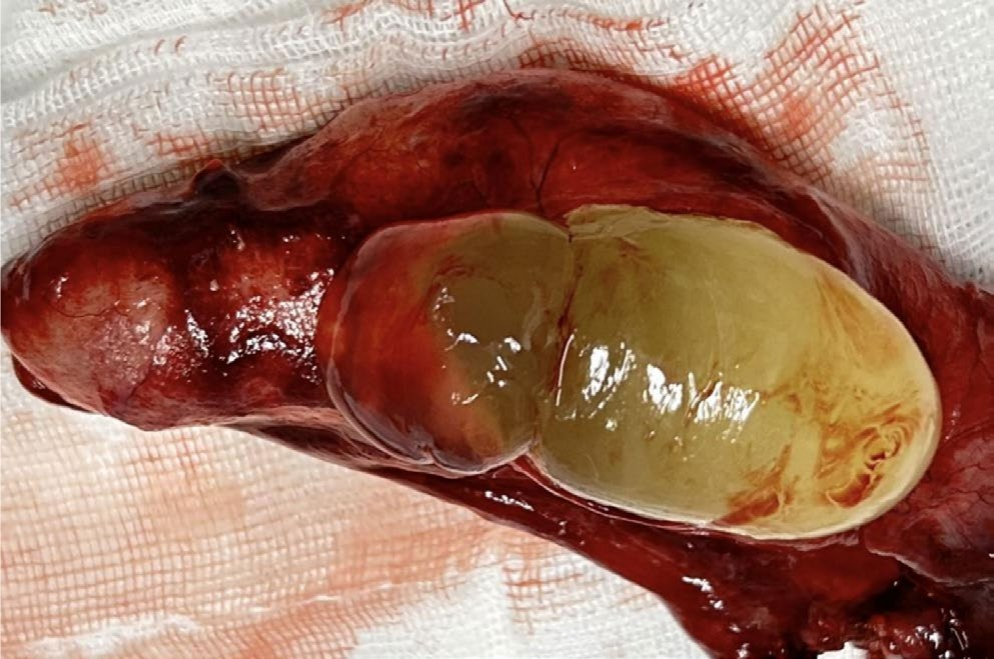
The appendix full of abundant transparent, yellow mucin.

**FIGURE 5:**
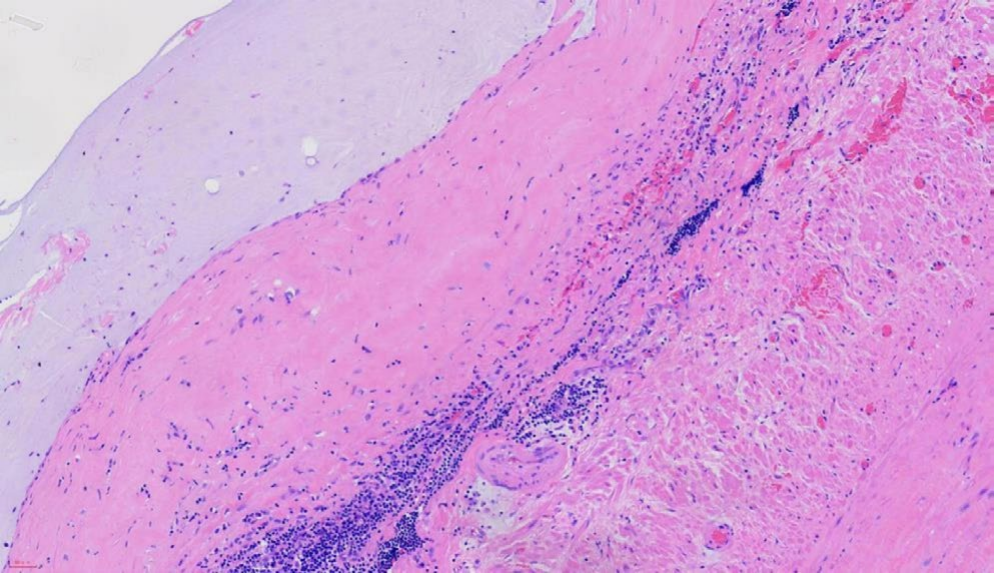
Mucous columnar epithelium lined the lumen of the appendiceal cavity, with peripheral inflammation, chronic inflammatory cell infiltration, and giant cell reaction. Light‐to‐moderate heteromorphic cells were observed (400× magnification).

The histologic feature of LAMN, the capsule of the appendix was complete, and abundant transparent yellow mucin existed in the capsule (Figures 2, 3, 4). Microscopic examination revealed that the mucous columnar epithelium lined the lumen of the appendiceal cavity, with acute inflammatory cell infiltration, a giant cell reaction, and mild to moderate heteromorphic cells observed. A negative margin was noted. The appendiceal epithelium was replaced by mucin‐columnar glandular epithelium with low‐grade dysplasia (Figure 5). Immunohistochemistry showed CK20 (+), CDX‐2 (+), CEA (+), Mucin‐4 (+), P53 (wild type), CK7 (−), Ki‐67 (hot spot area about 20%+), smoothelin (−), PTEN (−), Pax‐8 (−), SATB2 (+), and WT1 (−).

## 4 Conclusion and Follow‐Up Results

Tumor biomarkers of CEA and CA19‐9 in the blood, along with CT scans every 3 months, were monitored during the follow‐up process; tumor biomarkers of CEA, CA19‐9, or CA125 levels were measured in the blood per 3 months. These biomarkers' baseline values are CEA < 5, CA19‐9 < 36, or CA125 < 35. These biomarkers showed no abnormalities. Even now, the patient remained healthy, with no evidence of recurrence.

## 5 Discussion

Patients with an LAMN are often misdiagnosed with a tumor originating from the right ovary. The diagnosis of LAMN is challenging, particularly in female patients. This is because primary appendiceal and ovarian mucinous neoplasms share common atypical clinical and imaging findings. It can be challenging to diagnose LAMN in patients with acute appendicitis, particularly when the tumors originate in the lower right abdomen of young female patients. A colonoscopy and abdominal CT scan should help confirm the diagnosis. Blood levels of the tumor biomarkers CA19‐9, CEA, and CA125 indicate an increased risk of tumor or cancerous conditions, as well as the need for closer monitoring or intervention [2, 3].

LS is a reliable and safe strategy for appendiceal mucocele [4]. It is an essential tool for diagnosing and treating abdominal tumors, including appendiceal mucinous neoplasm and ovarian cystic neoplasm [5]. In our case, the tumor was located in the body of the appendix, and the root margin was negative. After consulting with our group during the procedure, we opted for a laparoscopic appendectomy.

There are many advantages of LS when contrasted with laparotomy [6]. It is not only less invasive but also useful as an intraoperative diagnostic tool for cases without a definitive preoperative diagnosis; it is a primary treatment option for appendiceal tumors. This assertion is substantiated by a comparative analysis with open surgical procedures [5]. A diagnostic exploration is under magnification; the whole abdominal cavity can be examined to find the appendix and assess for peritoneal dispersion. LS may reduce stress reactions, alleviate postoperative discomfort, and shorten hospital stays, suggesting a promising role in enhancing patients' well‐being and reducing medical expenses [7].

During the initial operation, the appendix and the fat tissue around its mesentery must be removed. To prevent the formation of a diffuse mucinous tumor, we must carefully resect the appendix with the use of laparoscopic vision. In the course of performing an open appendectomy, the area surrounding the incision is the only part that can be subject to visual inspection for the presence of any potential metastatic lesions. Minimal pneumoperitoneum pressure was utilized throughout the procedure to ensure the safety of patients undergoing laparoscopic appendectomy treated with a specimen retrieval bag. The goal is to prevent the disease from progressing to PMP by removing the appendix and the mesoappendix [8].

The decision to perform a right hemicolectomy was guided by the principle of depending on negative margins. Preserving the function of the ascending colon and the ileocecal valve is imperative to improve the patient's quality of life. The tumor is confined to the appendix, and the surgical intervention of appendectomy is deemed sufficient in itself [9]. Right hemicolectomy is recommended when patients with a positive margin, appendiceal rupture, mucin, or cells outside the appendix have a significantly higher possibility of developing PMP [2, 10, 11, 12, 13], where diffused intraperitoneal mucinous tumors and free mucin have a high recurrence rate and malignant spread [14, 15]. When considering right hemicolectomy, it is important to consider the histologic grade of the tumor, invasion through the muscular propria, size (> 2 cm), and involvement of the peri‐appendiceal area [16]. Morano suggested that the initial course of therapy is an appendectomy and that the pathological diagnosis should decide whether more resection is required [17] (Table 1).

**TABLE 1:** Appendiceal mucinous neoplasm surgical treatment strategy.

Clinical characteristics	Surgical recommendations	Critical basis
Low‐grade appendiceal mucinous neoplasm		
The tumor is complete and unruptured, does not involve the root of the appendix, and the margin is negative	Appendectomy	There is no risk of infiltration or implantation, and complete resection is sufficient for cure
Neoplasm rupture/involvement of the cecal wall, or positive root margin	Right hemicolectomy	Make sure there's no mucus or tumor cells left behind, and that the margins are negative
High‐grade tumor (HAMN/mucinous adenocarcinoma)		
High‐grade histology involves the peri‐appendiceal area, a larger size of more than 2 cm, or invades through the muscularis propria [16] Whether ruptured or involving the cecum	Routine right hemicolectomy	The principles of radical treatment for colon cancer: • Lymph node dissection in the affected area • Sufficient surgical margins • Resection of the affected intestinal segment
Peritoneal pseudomyxoma (PMP)		
Widespread appendiceal implantation or intestinal mucosal infiltration	Right hemicolectomy (Combined CRS + HIPEC)	Completely remove all visible lesions to achieve complete cytological eradication

The prognosis for LAMN is related to several factors, including serum tumor markers, the surgical procedure, and the pathology. Patients with LAMN of uncertain malignant potential who have negative margins and normal tumor marker levels are at lower risk for recurrence. To extend survival, LAMN with local peritoneal metastases is treated by cytoreductive surgery, hyperthermic intraperitoneal chemotherapy, and early postoperative intraperitoneal chemotherapy.

Our team was able to determine if the appendix or the ovary was the original location of the illness by using laparoscopy to treat a female patient with LAMN. The patient exhibited no indications of recurrence and demonstrated robust health following 4 years of observation. Laparoscopy facilitated the identification of laparoscopically treated LAMN patients, particularly for young women experiencing fixed abdominal discomfort in the lower right quadrant.

## Author Contributions

**Ping Xiao:** conceptualization, writing – original draft. **Tao Xu:** investigation, methodology. **Ming Zhou:** supervision, writing – review and editing. **ZhenYu Lei:** project administration, validation.

## Funding

The authors have nothing to report.

## Consent

Written informed consent was obtained from the patient for the publication of this case report and any accompanying images. A copy of the written consent is available for review by the Editor‐in‐Chief of this journal.

## Conflicts of Interest

The authors declare no conflicts of interest.

## Data Availability

There is no data in this article, Thanks!.
